# Supplementary figures and images for: Predicting 30-day hospital readmissions using artificial neural networks with medical code embedding
Source: PLoS One. 2020 Apr 15;15(4):e0221606. doi: 10.1371/journal.pone.0221606 (PMC7159221; doi:10.1371/journal.pone.0221606)

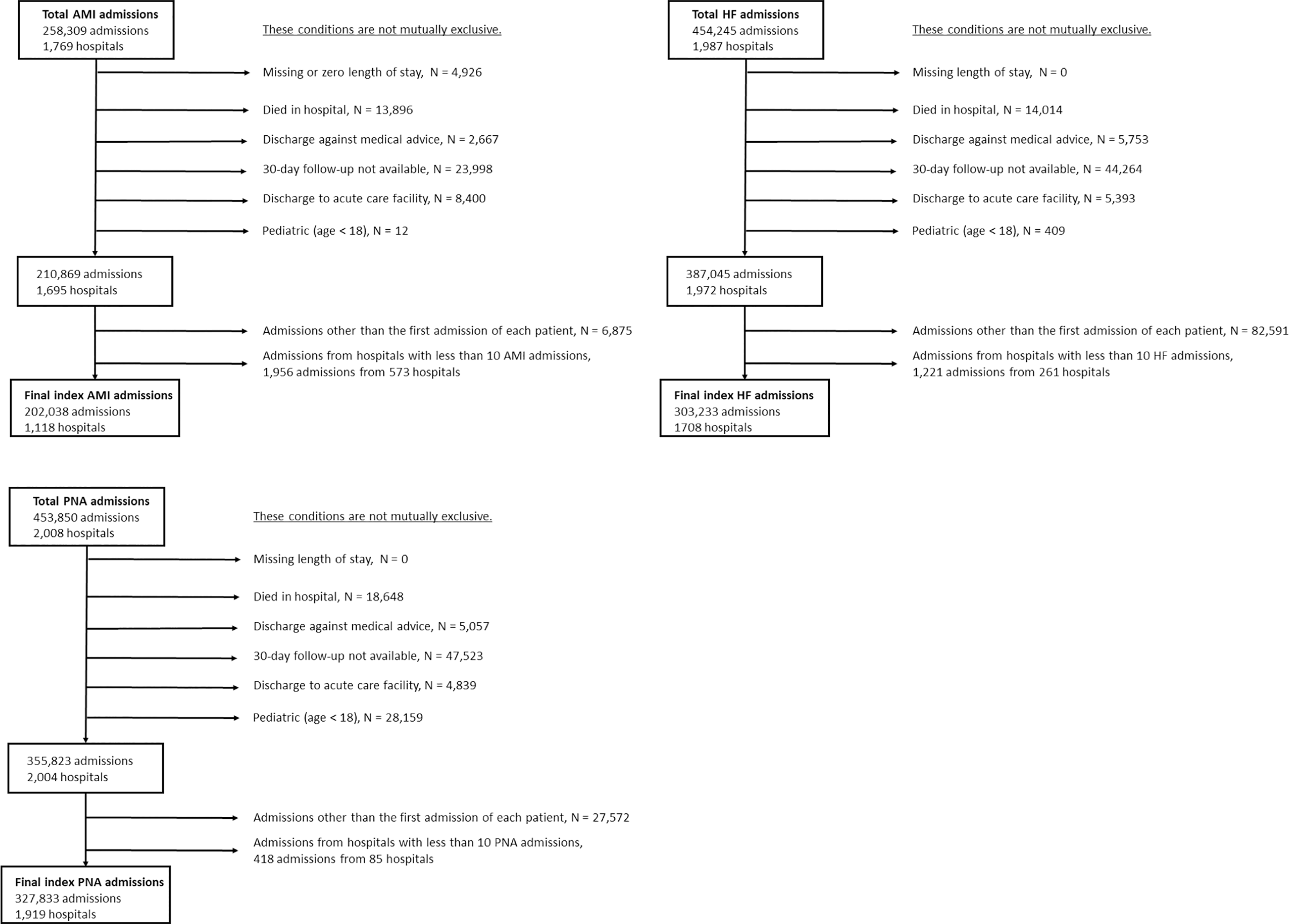

Supplement: S1 Fig — Cohort selection for AMI, HF, and PNA are shown in (a), (b) and (c) respectively. (TIFF) [file pone.0221606.s002.tiff]

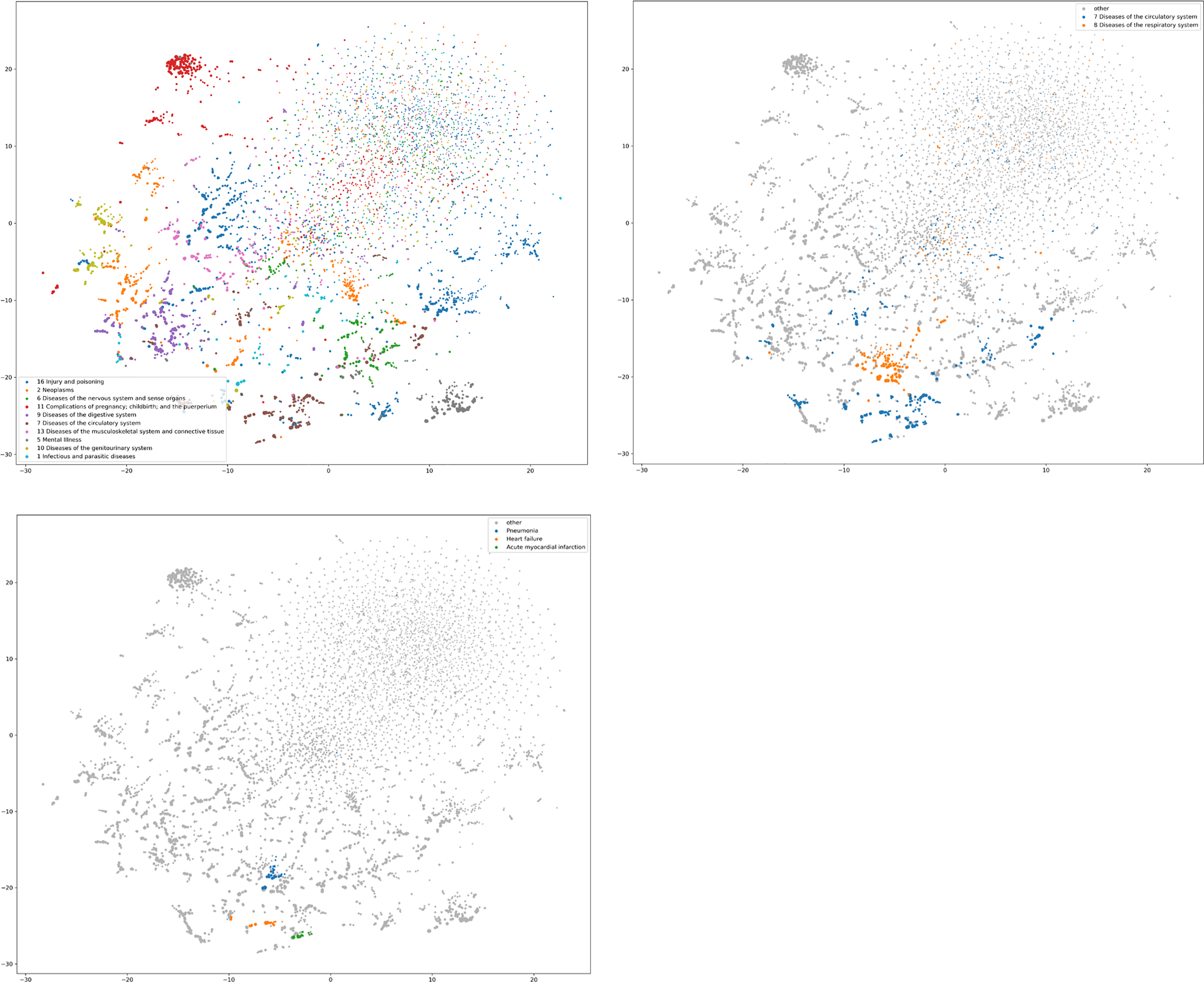

Supplement: S2 Fig — This visualization was done using t-SNE. Each point represents a diagnosis code (disease). The size of the points represents the prevalence of that code. (a) The points are coloured by the Clinical Classifications Software (CCS)10 level 1 categories of the multi-level classification system. The frequent codes with the same CCS level 1 categories form clusters, while the infrequent codes form a “cloud” without a clear pattern. (b) As examples, two CCS level 1 categories, “7 Diseases of the circulatory system” and “8 Diseases of the respiratory system” are highlighted in the visualization, with all other diseased represented in grey. (c) The principal diagnosis codes as the inclusion criterion of the three cohorts, acute myocardial infarction, congestive health failure and pneumonia are highlighted. (TIFF) [file pone.0221606.s003.tiff]

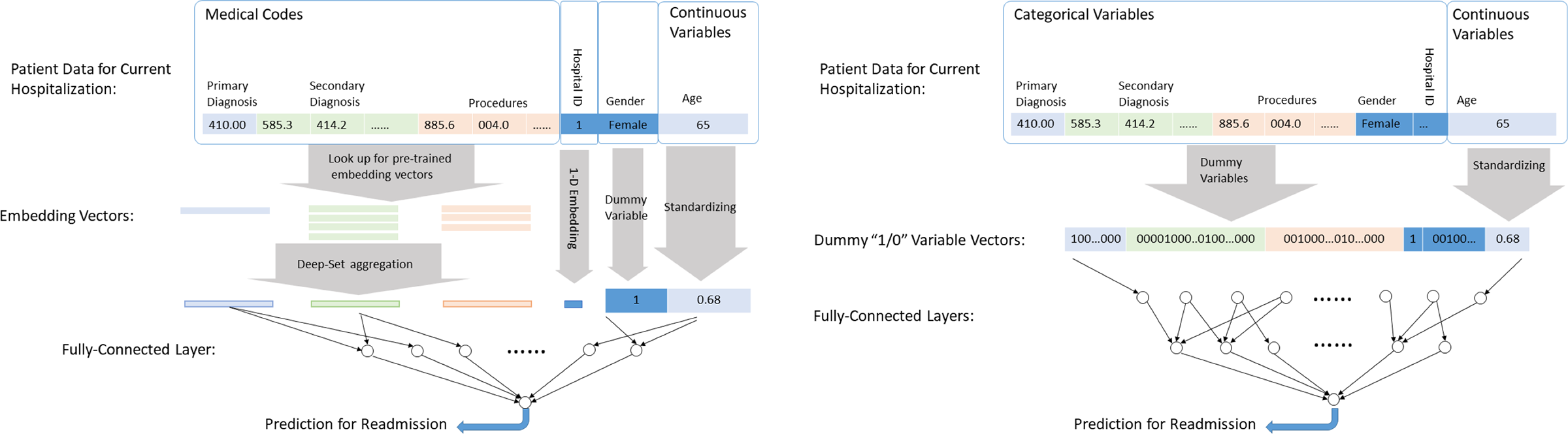

Supplement: S3 Fig — (a) Feed-forward neural network ANN model. (b) Medical code embedding deep set architecture model. This model looks up the medical code embedding of each ICD-9 codes that are pretrained by the GloVe model, and aggregates variable number of secondary diagnosis and procedure codes into a final representation vector using the deep set architecture. (TIFF) [file pone.0221606.s004.tiff]

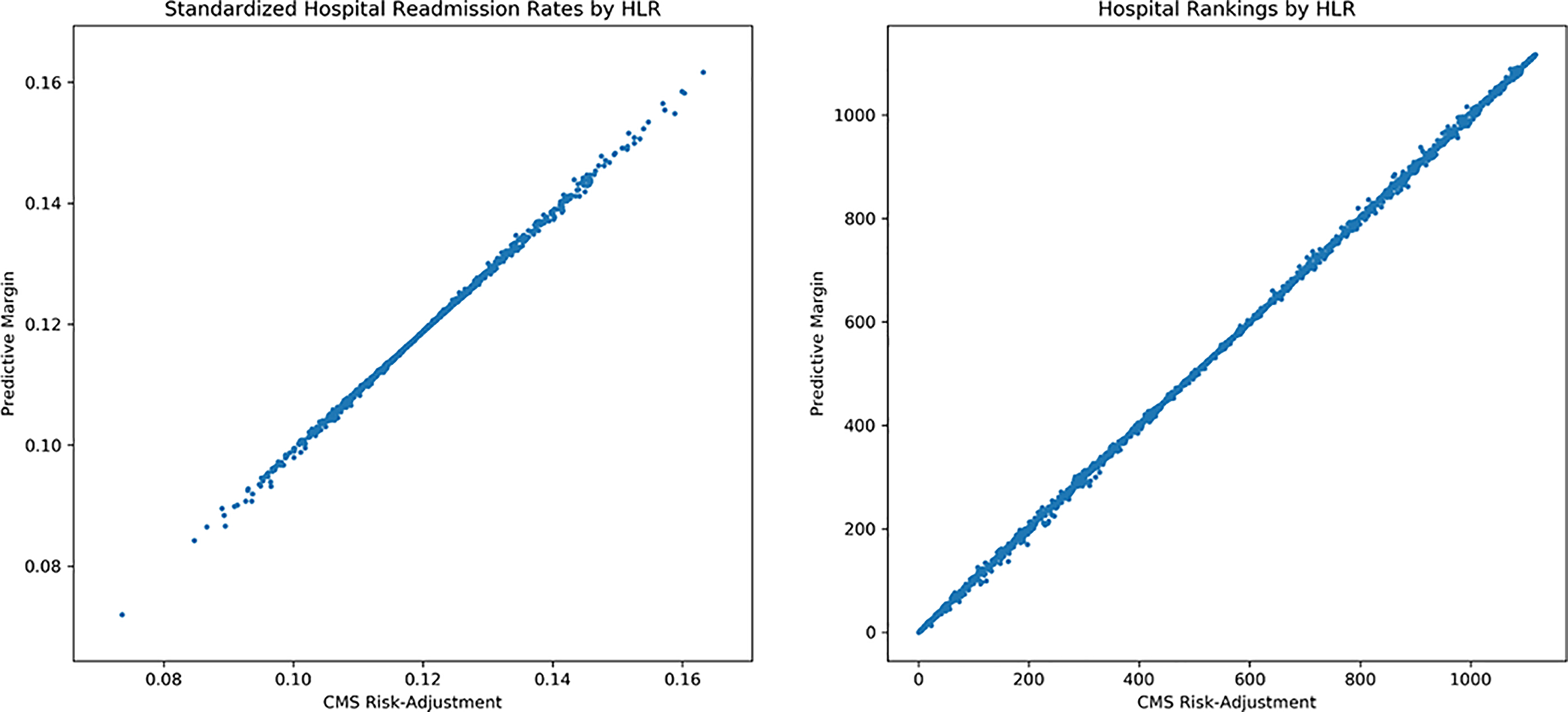

Supplement: S4 Fig — The plots shows the risk-standardized hospital readmission rates and the hospital rankings calculated by the two risk-adjustment methods, predicted over expected readmission rate ratio (e.g., the method employed by the Centers for Medicare & Medicaid Services [CMS]) and predictive margins, for the hierarchical logistic regression (HLR) model on the acute myocardial infarction cohort. (TIFF) [file pone.0221606.s005.tiff]
