# Supplementary material for: Predicting 30-day hospital readmissions using artificial neural networks with medical code embedding
Source: PLoS One. 2020 Apr 15;15(4):e0221606. doi: 10.1371/journal.pone.0221606 (PMC7159221; doi:10.1371/journal.pone.0221606)
Supplement: S1 Table — (DOCX) [file pone.0221606.s006.docx]

**STable 1. Summary statistics of ICD-9CM diagnosis and procedure codes for each cohort.**

|  |  | | **Acute Myocardial Infarction** | **Heart Failure** | **Pneumonia** |
| --- | --- | --- | --- | --- | --- |
| Principal diagnosis | No. of distinct codes | | 20 | 24 | 32 |
| Secondary diagnosis | No. of distinct codes | | 5,614 | 6,032 | 6,690 |
|  | Frequency  Quartiles | 25% | 2 | 2 | 3 |
|  |  | 50% | 7 | 10 | 12 |
|  |  | 75% | 49 | 69 | 77 |
|  |  | maximum | 152,602 | 237,572 | 143,155 |
| Procedure | No. of distinct codes | | 1,295 | 1360 | 1,443 |
|  | Frequency  Quartiles | 25% | 1 | 1 | 1 |
|  |  | 50% | 4 | 4 | 4 |
