# Supplementary material for: Predicting 30-day hospital readmissions using artificial neural networks with medical code embedding
Source: PLoS One. 2020 Apr 15;15(4):e0221606. doi: 10.1371/journal.pone.0221606 (PMC7159221; doi:10.1371/journal.pone.0221606)
Supplement: S1 Information — (DOCX) [file pone.0221606.s007.docx]

# Supplementary Information

This supplement describes additional details and results from the paper by Wenshuo Liu et al. entitled “Predicting 30-day Hospital Readmissions Using Artificial Neural Networks with Medical Code Embedding”. List of elements in this supplement:

- Details of the Artifiial Neural Network Models.
- Calculation of Risk-Standardized Hospital Readmission Rates.
- SFigure 1. Flow diagrams of the cohort creation for acute myocardial infarction (AMI), congestive health failure (HF), and pneumonia (PNA).
- SFigure 2. Architectures of Artificial Neural Network (ANN) Models.
- SFigure 3. Visualization of embedding vectors of the principal diagnosis codes in two dimensions.
- SFigure 4. Comparison of risk-adjustment methods.
- STable 1. Summary statistics of ICD-9CM diagnosis and procedure codes for each cohort.
- STable 2. The prediction accuracy of 30-day readmission for elder (65 years old or above) patients.

## Details of the XGBoost Models

XGBoost is an open-source software library1 which provides a gradient boosting2,3 framework in many programming languages under various operating systems. From the project description, it aims to provide a “Scalable, Portable and Distributed Gradient Boosting Library”4. We implemented the XGBoost model by the Python pacakage xgboost with the default parameter setting. We represented the ICD-9 diagnosis and procedure codes as dummy variables (1 if present, 0 if absent) and used them as the predictors in addition to age and gender. Due to the large number of distinct ICD-9 codes, and large cohort sizes in our problem, this model suffers from large consumption of memory. In our implementation, the XGBoost model required 96 Gb of memory for the pneumonia cohort (which has the largest sample size), and had to run on a special computational server.

## Details of the Artificial Neural Network Models

The network architectures of the two artificial neural network (ANN) models are illustrated in SFigure 2. Details of the methodology are explained in the following paragraphs.

## Multi-Space GloVe

We modified the original Global Vectors for Word Representation (GloVe) model5 to fit unique apsects of our particular problem around medical code embeddings. Specifically, we considered the ICD-9 principal diagnosis codes, the secondary diagnosis codes and the procedure codes to be three different categories of variables, and therefore embedded them into different spaces with dimensions 200, 200 and 50, respectively. These spaces are connected via matrix multiplications in the GloVe model, and the elements of the matrices are free parameters to be trained. In this way, we have freedom to adjust the embedding dimension according to the vocabulary size (number of distinct codes) and prevalence of the variable category. For example, the ICD-9 procedure codes had a smaller vocabulary size and much lower average prevalence, compared with the diagnosis codes, so we chose a smaller dimension for them. Our experiments showed that embedding of medical codes into multiple spaces improved prediction accuracy over the original GloVe model.

## Deep Set Architecture

Because the numbers of secondary diagnosis and procedure codes may differ for different patients, and the model should be invariant to the ordering of the diagnosis and procedure codes, we used a deep set architecture6 to aggregate embeddings of these codes. During implementation, we used a fully-connected layer as a transformation to map the medical code embeddings into a latent variable space and then do a simple summation in that latent space. Each category of ICD-9 codes (principal diagnosis, secondary diagnosis or procedure codes) has its own transformation. Our experiments showed that a deep set architecture improved prediction accuracy than simple aggregation functions such as summation or max pooling.

## ANN Model Training

The two ANN models in this work have several hyper-parameters to be tuned, including the dimensions of the medical code embeddings, the width of the fully-connected layers, learning rate, batch size, strength of dropout, among others. All the hyper-parameters were chosen through cross-validation to give the best prediction accuracy. These experiments are available from the authors. The ANN model using feed-forward neural network was trained end-to-end. When training the medical code embedding deep set architecture model, we first froze the pre-trained embedding and tuned the network weights, then we allowed the embedding to vary and fine-tuned the whole network with a smaller learning rate. Both of the ANN models were trained by the Adam algorithm.7 We used dropout as regularization.8 During training, we monitored the area under the curve (AUC) for Reiver Operating Characteristic on the validation set, and saved the model with the best AUC.

## Calculation of Risk-Standardized Hospital Readmission Rates

To assess for differences in hospital ranking caused by the different models, we used predictive margins to calculate risk-standardized hospital readmission rates, as this method can be applied to any prediction model (including ANN models). However, we recgonize that the Centers for Medicare & Medicaid Services (CMS) and other healthcare policy-makers frequently use the predicted over the expected readmission rate ratio as the risk-standardization method, which only applies to the hierarchical logistic regression (HLR) models. Thus, we calculated the risk-standardized hospital readmission rates by the two methods on the HLR model to ensure comparability, and showed the results on the acute myocardial infarction cohort as an example in Supplementary Figure 4. The two methods yielded consistent risk-standardized hospital readmission rates and hospital rankings across all 3 conditions.

# References

1. Chen, T. & Guestrin, C. XGBoost: A Scalable Tree Boosting System. in *Proceedings of the 22nd ACM SIGKDD International Conference on Knowledge Discovery and Data Mining - KDD ’16* 785–794 (ACM Press, 2016). doi:10.1145/2939672.2939785

2. Friedman, J. H. Greedy function approximation: A gradient boosting machine. in (2001). doi:10.1214/aos/1013203451

3. Mason, L., Baxter, J., Bartlett, P. L. & Frean, M. R. Boosting Algorithms as Gradient Descent. in *Advances in Neural Information Processing Systems 12* (eds. Solla, S. A., Leen, T. K. & Müller, K.) 512–518 (MIT Press, 2000).

4. XGBoost Documentation — xgboost 0.90 documentation. Available at: https://xgboost.readthedocs.io/en/latest/index.html. (Accessed: 22nd May 2019)

5. Pennington, J., Socher, R. & Manning, C. D. GloVe: Global Vectors for Word Representation. in *Empirical Methods in Natural Language Processing (EMNLP)* 1532–1543 (2014).

6. Zaheer, M. *et al.* Deep Sets. in *Advances in Neural Information Processing Systems 30* (eds. Guyon, I. et al.) 3391–3401 (Curran Associates, Inc., 2017).

7. Kingma, D. P. & Ba, J. Adam: A Method for Stochastic Optimization. *arXiv:1412.6980 [cs]* (2014).

8. Srivastava, N., Hinton, G., Krizhevsky, A., Sutskever, I. & Salakhutdinov, R. Dropout: A Simple Way to Prevent Neural Networks from Overfitting. *Journal of Machine Learning Research* **15**, 1929–1958 (2014).

9. Maaten, L. van der & Hinton, G. Visualizing Data using t-SNE. *Journal of Machine Learning Research* **9**, 2579–2605 (2008).

10. Elixhauser, A., Steiner, C. & Palmer, L. Clinical Classifications Software (CCS), 2015. *U.S. Agency for Healthcare Research and Quality*

**SFigure 1. Flow diagrams of the cohort creation for acute myocardial infarction (AMI), congestive health failure (HF), and pneumonia (PNA).**

**a**


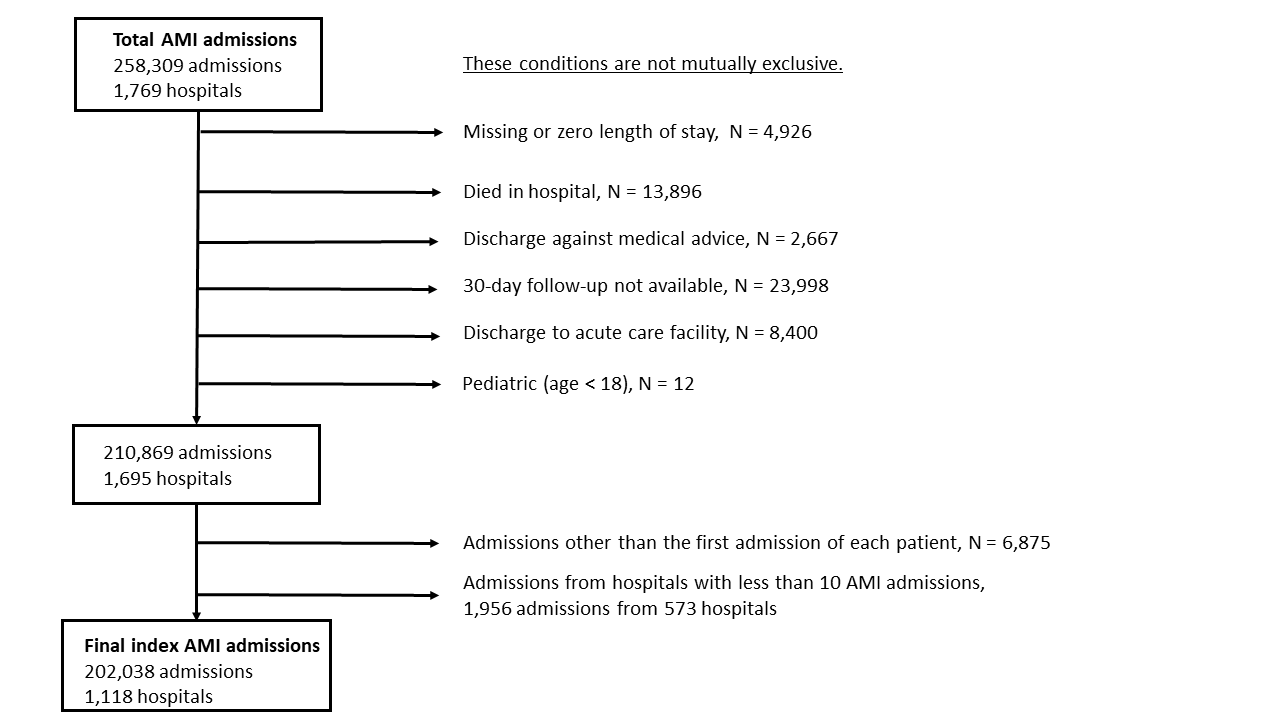


**b**


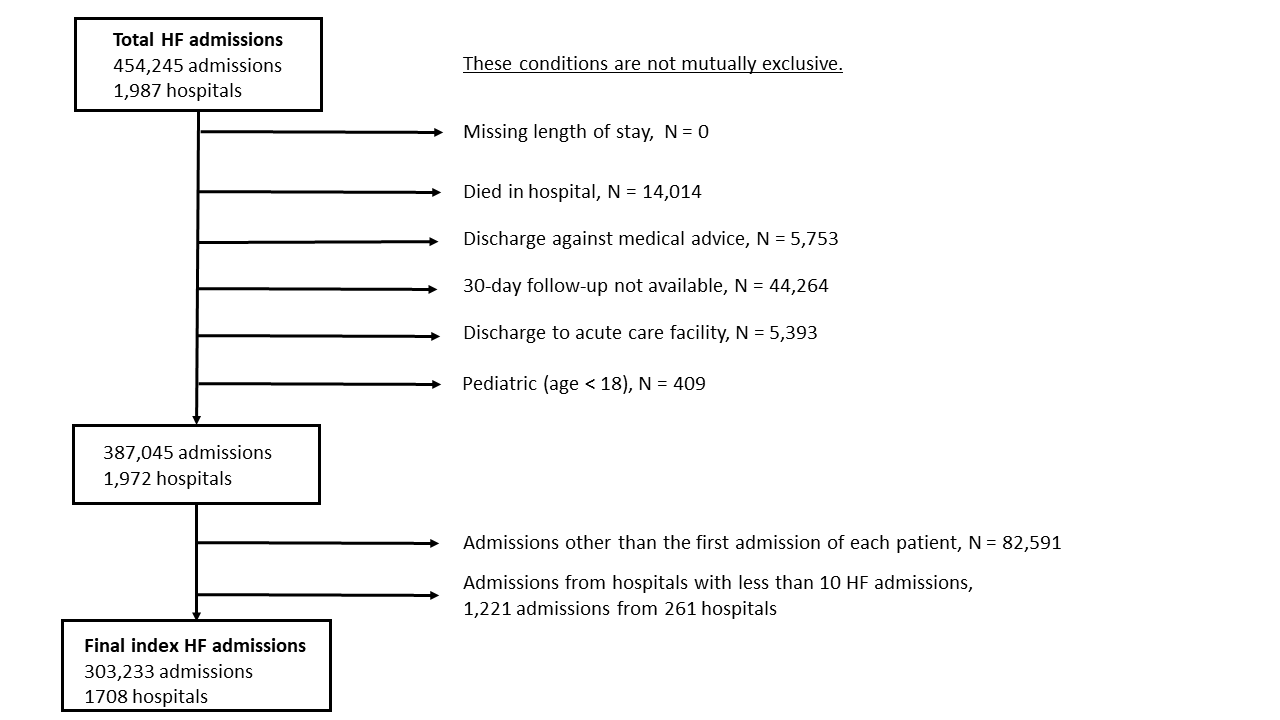


**c**


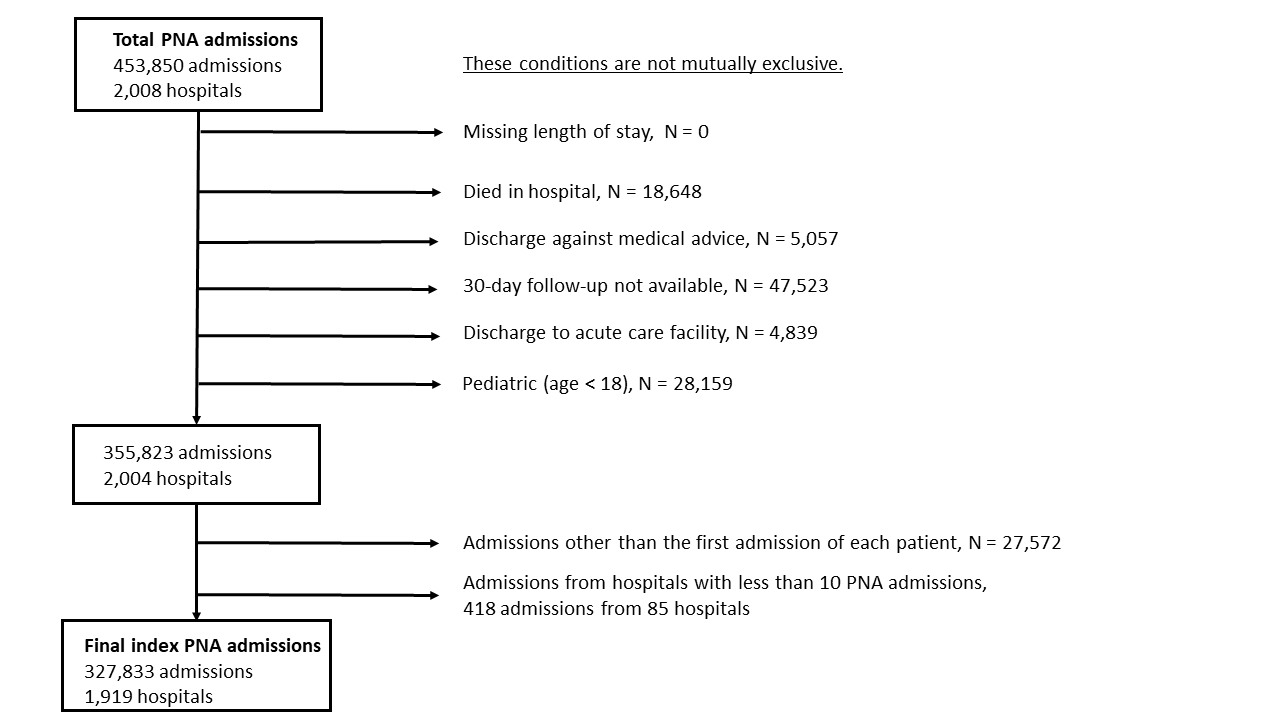


**SFigure 2. Visualization of embedding vectors of the principal diagnosis codes in two dimensions.**

This virsualization was done by t-SNE9. Each dot represents a diagnosis code (disease). The size of the dots represents the prevalence of that code. (a) The dots are coloured by the Clinical Classifications Software (CCS)10 level 1 categories of the multi-level classification system. The frequent codes with the same CCS level 1 categories form clusters, while the infrequent codes form a cloud without a clear pattern since there is not enough data to learn their exact positions. It demonstrates the rationale of medical code embedding: related diseases are closer. (b) As examples, two CCS level 1 categories, “7 Diseases of the circulatory system” and “8 Diseases of the respiratory system” are highlighted in the visualization, with all other diseased represented by grey. (c) The principal diagnosis codes as the inclusion criterion of the three cohorts, acute myocardial infarction, congestive health failure and pneumonia are highlighted. They all form very tight clusters, verifying the rationale of the cohort creation.

**a.**


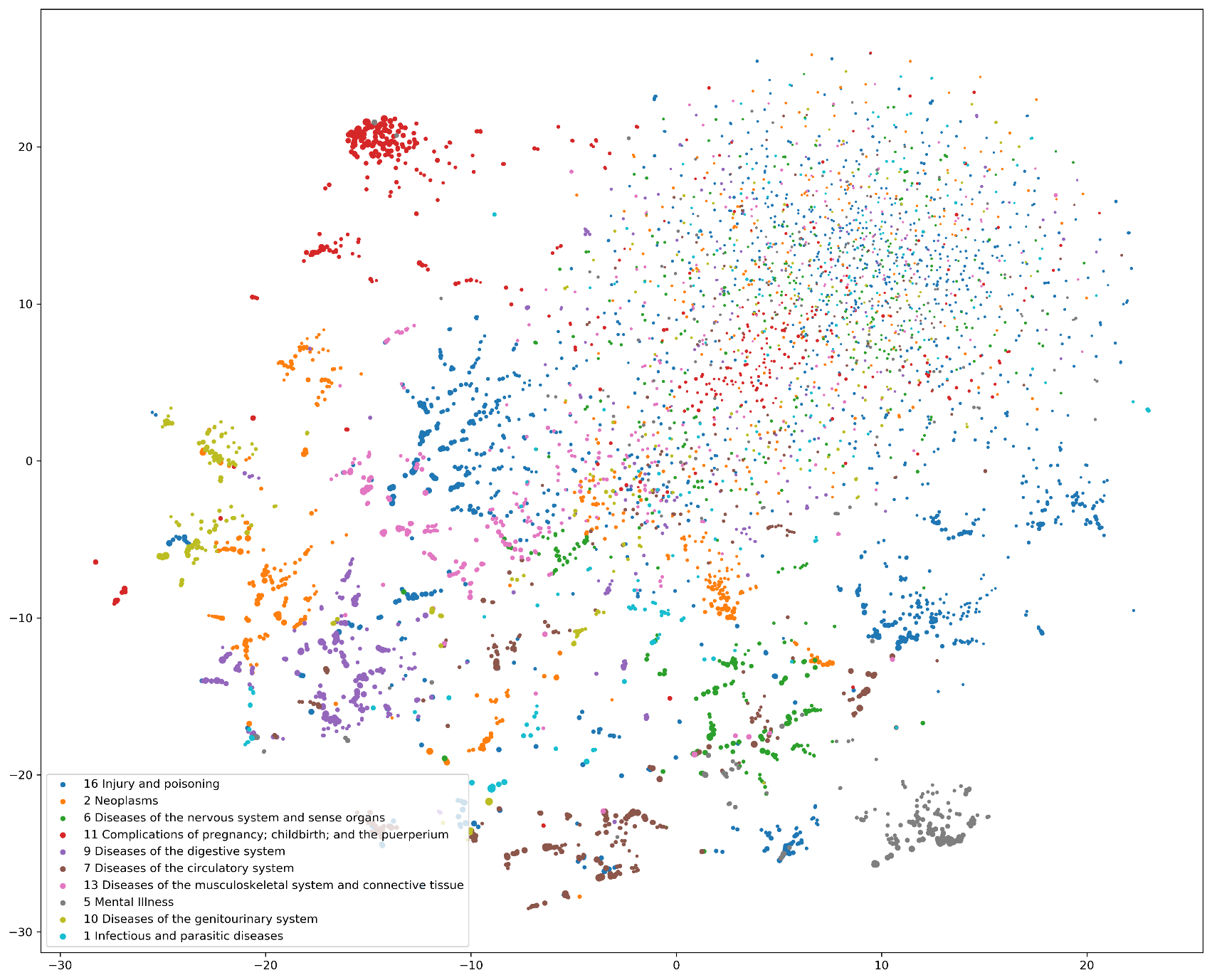


**b.**


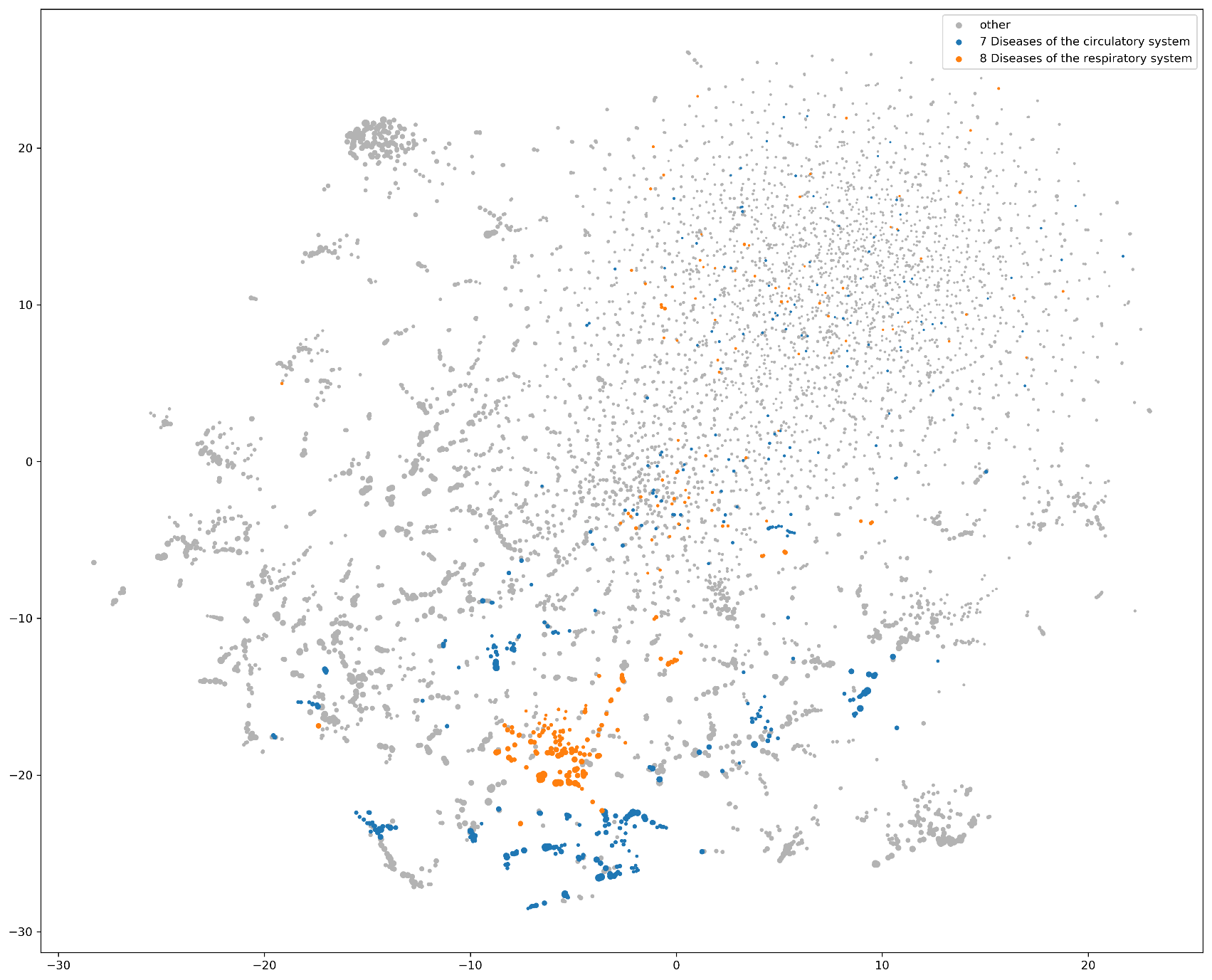


**c.**


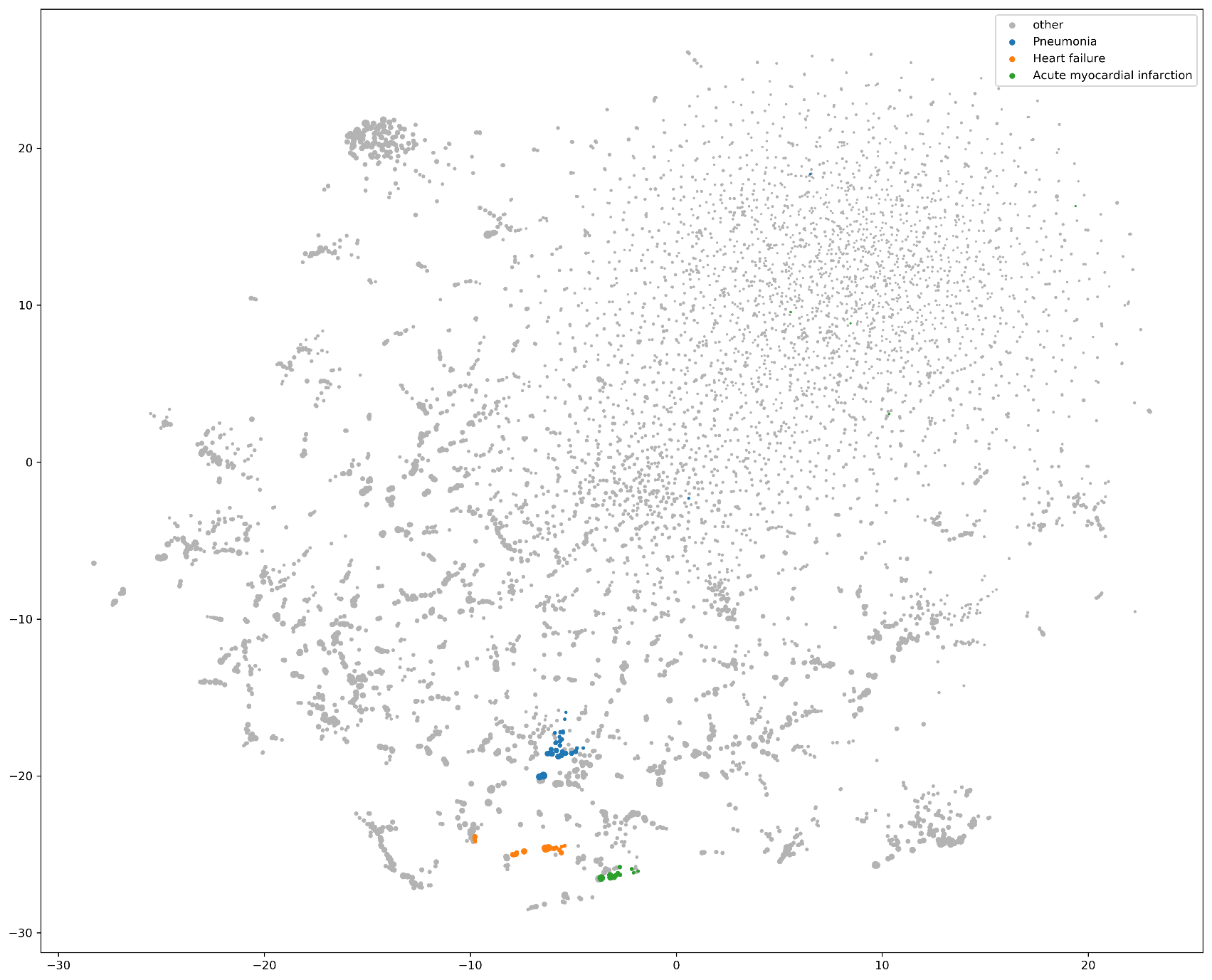


**SFigure 3. Architectures of Artificial Neural Network (ANN) Models.**

(a) Feed-forward neural network ANN model. In this model, the ICD-9 diagnosis and procedure codes are represented as dummy variable vectors consisting of 0s and 1s, with 1 indicating the existence of certain diseases. The gender and hospital ID, as categorical variables, were treated in the same way. The dummy variable vectors are concatenated with standardized age, and then fed into a feed-forward neural network with two fully connected layers, and finally give the probability score of readmission. (b) Medical code embedding deep set architecture model. This model looks up the medical code embedding of each ICD-9 codes that are pretrained by the GloVe model, and aggregates variable number of secondary diagnosis and procedure codes into a final representation vector using the deep set architecture. The hospital ID is embedded as a 1-dimensional variable, playing a similar role as the hospital-specific intercept in the hierarchical logistic regression model. The concatenation of the final representation vectors and with gender and age goes through one fully connected layer to give the probability score of readmission.

**a**


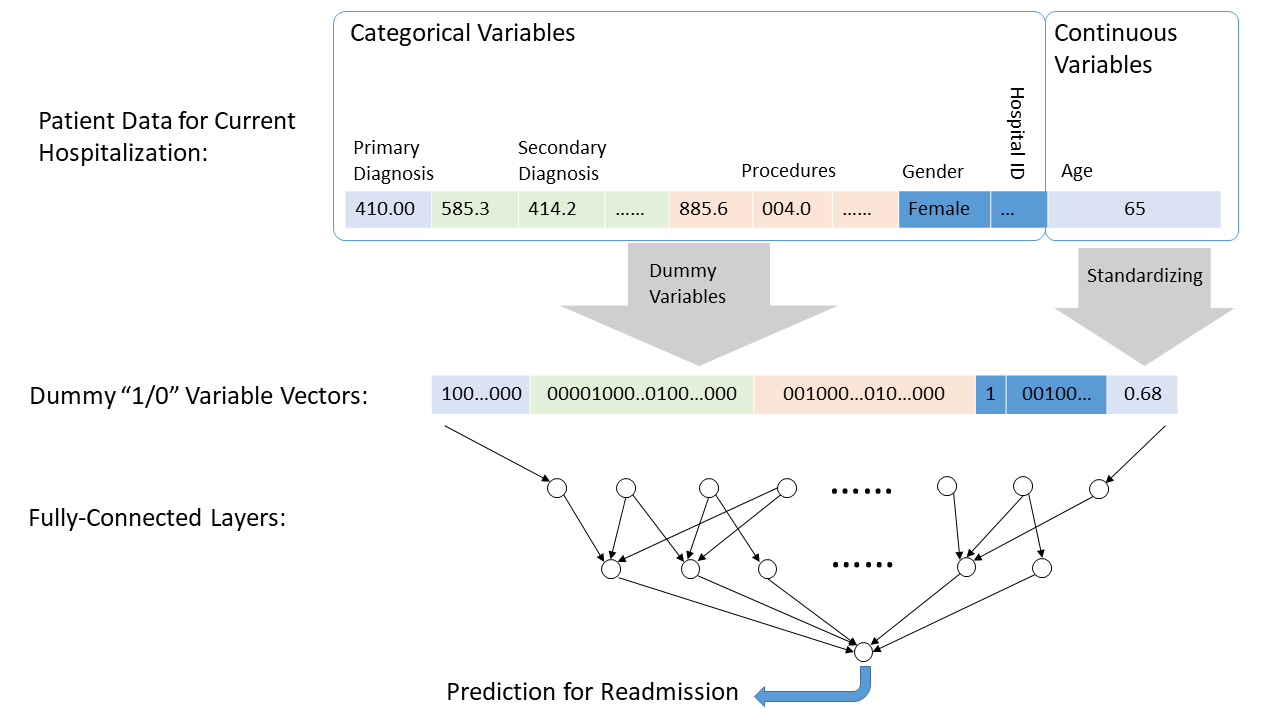


**b**


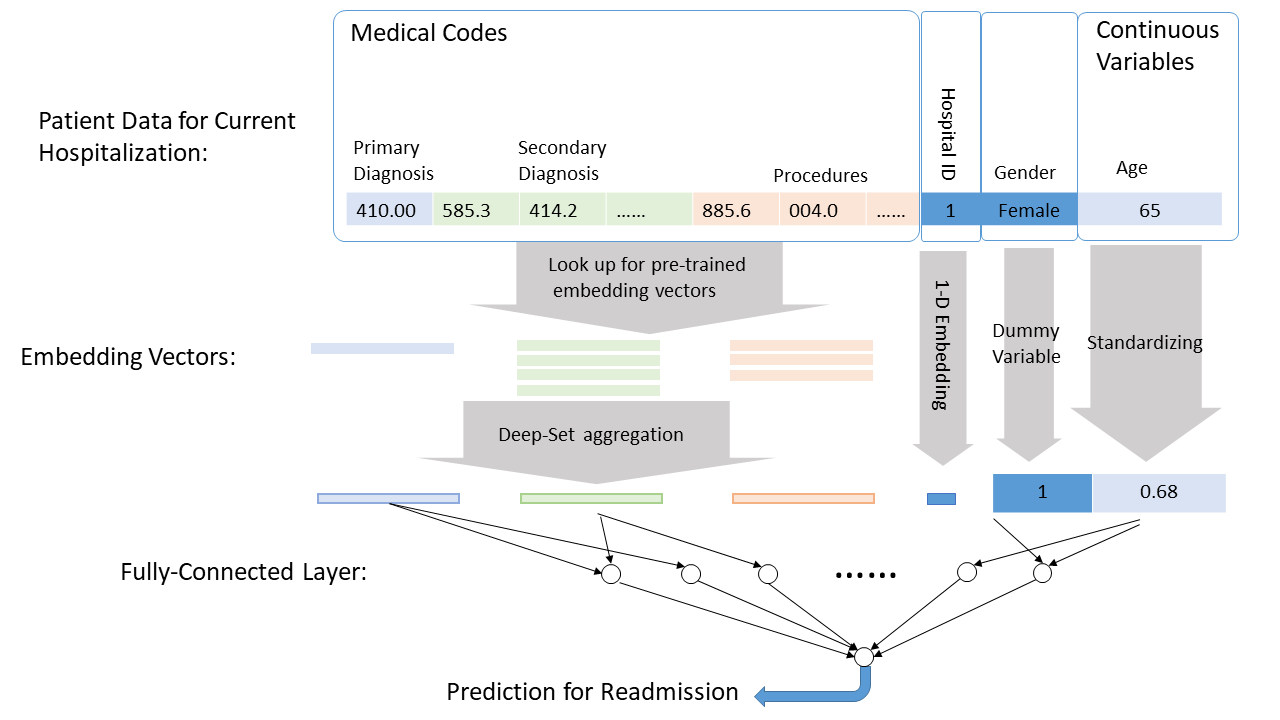


**SFigure 4. Comparison of risk-adjustment methods.**

The plots shows the risk-standardized hospital readmission rates and the hospital rankings calculated by the two risk-adjustment methods, predicted over expected readmission rate ratio (e.g., the method employed by the Centers for Medicare & Medicaid Services [CMS]) and predictive margins, for the hierarchical logistic regression (HLR) model on the acute myocardial infarction cohort.


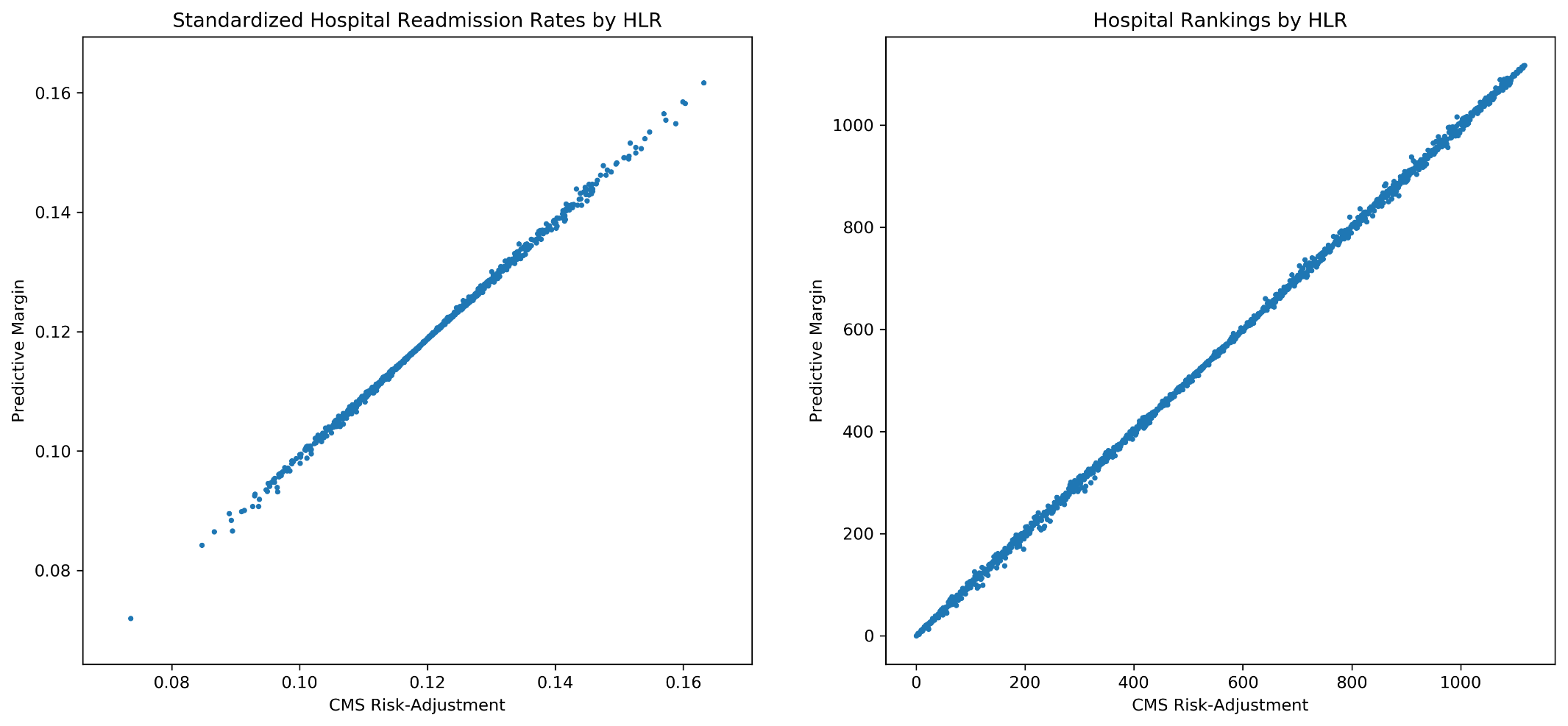


**STable 1. Summary statistics of ICD-9CM diagnosis and procedure codes for each cohort.**

|  |  | | **Acute Myocardial Infarction** | **Heart Failure** | **Pneumonia** |
| --- | --- | --- | --- | --- | --- |
| Principal diagnosis | No. of distinct codes | | 20 | 24 | 32 |
| Secondary diagnosis | No. of distinct codes | | 5,614 | 6,032 | 6,690 |
|  | Frequency  quartiles | 25% | 2 | 2 | 3 |
|  |  | 50% | 7 | 10 | 12 |
|  |  | 75% | 49 | 69 | 77 |
|  |  | maximum | 152,602 | 237,572 | 143,155 |
| Procedure | No. of distinct codes | | 1,295 | 1360 | 1,443 |
|  | Frequency  quartiles | 25% | 1 | 1 | 1 |
|  |  | 50% | 4 | 4 | 4 |
|  |  | 75% | 22 | 31 | 19 |
|  |  | maximum | 141,375 | 27,406 | 17,615 |

**STable 2. The prediction accuracy of 30-day readmission for elder (65 years old or above) patients.**

The prediction accuracy was assessed by the area under the curve for Receiver Operating Characteristic (AUC) on the three cohorts. We compared the four models: the hierarchical logistic regression, XGBoost, the feed-forward neural networks, and the medical code embedding Deep Set architecture model.

| **Methods** | **Acute Myocardial Infarction** | **Heart Failure** | **Pneumonia** |
| --- | --- | --- | --- |
| Hierarchical Logistic Regression | 0.639 (0.635, 0.642) | 0.580 (0.578, 0.583) | 0.605 (0.601, 0.609) |
| XGBoost | 0.666 (0.664, 0.668) | 0.602 (0.599, 0.605) | 0.635 (0.632, 0.638) |
| Feed-Forward Neural Networks | 0.667 (0.664, 0.670) | 0.604 (0.602, 0.606) | 0.639 (0.636, 0.641) |
| Medical Code Embedding Deep Set Architecture | 0.683 (0.680, 0.686) | 0.618 (0.616, 0.621) | 0.656 (0.653, 0.658) |
