# Supplementary material for: Predicting 30-day hospital readmissions using artificial neural networks with medical code embedding
Source: PLoS One. 2020 Apr 15;15(4):e0221606. doi: 10.1371/journal.pone.0221606 (PMC7159221; doi:10.1371/journal.pone.0221606)
Supplement: Attachment — Submitted filename: PLOS-One-Response_Letter 12162019.docx [file pone.0221606.s008.docx]

**REVIEWER COMMENTS**

*Reviewer #1: This manuscript (ms) reports an Artificial Neural Network (ANN) based machine learning system for predicting 30-day hospital readmissions. The results show that including medical code embedding improves performance (AUC). The ms is well-written and well-organized.*

**Response:** We appreciate the positive comments.

*My concerns are:
1. The exclusion criterion #8 (page 6, line 172) says "with less than 10 admission". Why this was used as a criterion?*

**Response:** We used this criterion to eliminate hospitals that do not care for patients with these conditions frequently. For example, some of these facilities may include non-acute care hospitals or small rural facilities (e.g., critical access hospitals). Given that such facilities are not generally considered a part of typical quality assurance or performance measurement programs for readmissions, we were not interested in these facilities. We have added this comment to page 5 (bottom) in order to clarify our reasoning.

*2. It would be nice if the authors can provide some explanation why ANN models provide more accurate prediction of readmission and generate risk-standardized hospitalization rates.*

**Response:** This is a very good suggestion. We have added a sentence concisely explaining why the ANN trained on embeddings may have reached a higher performance to page 11 (top of page). We are careful not to claim that the risk-standardization scores based on ANNs are *better*, per se. We write:

*“One possible explanation for this performance increase is that the embeddings capture the co-occurrence relationship between diagnosis codes, which is not enjoyed by the other three models, and the ANN is able to learn non-linear mapping patterns between the embeddings and the outcome. ”*

Our claim is that the differences in final scores are large enough to warrant our attention, especially since the ANN model seems to explain hospital readmission more accurately than traditional approaches. We also have added a clarifying sentence to page 13 (middle of page).

*3. Table 3 was prepared by grouping the data at top 20%, middle 60%, and bottom 20%. Why these ranges were chosen? Is there any scientific reasoning?*

**Response:** This is a good question, thank you. These ranges (quintiles) divide the number of facilities into five equal partitions, which are equivalently two deciles each. Quintiles are common for summarizing large-scale population income statistics, economic statistics, and healthcare statistics since they provide an efficient way to communicate about relatively more mass in the tails than a decile does. We clump the middle quintiles because we are most interested in showing big picture changes. In our work, we have found that they provide an easy way to communicate large-scale changes in hospital rankings between the two scoring paradigms, *specifically* for those hospitals that moved to or from a tail (top 20% or bottom 20%). Ultimately, the choice in cut-off, is arbitrary. We feel that top and bottom 20% make sense for administrative data and is consistent with prior work by our group (Chan PS et al. JACC 2013;62:601; Thomas MP et al. PLOS One 2015;10:e0138251; Chan PS et al. JAMA Cardiology 2016;1:189).

*4. Is the improvement in performance significant? The results suggest marginal improvement.*

**Response:** Great question. This can, and should be, answered in two ways. First, we can say that the differences between the logistic regression model and the ANN trained on embeddings are significant because there is no overlap in confidence intervals around the AUC scores. For example, for the AMI cohort the logistic regression model AUC was 0.68 with a 95% confidence interval from 0.678 to 0.683. Meanwhile, the AUC of the ANN model trained on the embeddings reached 0.72 with a 95% confidence interval from 0.718 to 0.722. The lower-bound of the AUC from the ANN (0.718) does not overlap the confidence interval from the LR model at all, whose upper-bound is 0.683. This is the case for AUC scores for all conditions of interest. Thus, we can be reasonably sure that the AUC scores differ between models. Second, the magnitude of change that we find most interesting is not the small percentage gain in AUC, but rather the resulting changes in risk-standardization based on a different predictive method. Marginal (but significant) improvement over a logistic regression model, while worth noting, is most interesting because it results in changes to how we might stratify hospitals. Imagine the impact that a model that achieves 90% AUC might have on risk score standardization. Significance is not so easy to estimate here, but we feel that this warrants further investigation and suggests a potential role for models that incorporate an ANN.

*Reviewer #2: There are both pros and cons of the paper:
Pros:
1. The paper compared the performance of 4 different models on the application of predicting unplanned readmission based on a large database. The outcome is accurately and clearly defined.*

**Response:** Thank you!

*2. The result of the experiments seems good and it suggests the combination of ANN and medical code embedding as the top method for the readmission prediction.*

**Response:** We thank the reviewer for this feedback. We are also excited by these prospects.

*3. The author presented a very detailed introduction and discussion of ANN and embedding. Different models are well defined.*

**Response:** We thank the reviewer.

*Cons:
1. Some essential details of the experiments are missing. E.g. What are the parameters of your ANN and Xgboost and how did you tune them. What's the structure of your ANN? How many hidden neurons? What are the features you put in and how they are selected? These parameters are highly related to the performance.*

**Response:** The reviewer raises concerns about training details of the classifiers reviewed. We appreciate this feedback. We have moved some descriptive material from the supplemental material to the manuscript in order to make experiments clearer. Further, we explicitly motivate the simplicity of both the feed-forward ANN and the XGBoost models in the context of our study.

Specifically, the reviewer asks whether the choice of particular hyper-parameters and features is highly related to model performance. The answer is that choice of model parameters and features always have an impact on results. We have attempted to present our methods to a semi-technical audience (the absence of formulas is important). However, the reader feels that more detail would be helpful.

Each of the models is discussed below. Changes to the manuscript are documented here as well:

1. We implemented the logistic regression model using the R function `glmer()`. We have specified the exact function used in the manuscript (page 7, top of page). This function allows for the specification of many parameters. We limited our parameters and features to defaults and chose widely used commodity data to implement a reasonable baseline representing current practices in risk standardization. Besides default parameters we have specified an optimizer (`nloptwrap`) and the nAGQ parameter (`nAGQ=7`). Increase beyond 1 (the default) of nAGQ parameter results in greater accuracy in log-likelihood. All other parameters were defaults for logistic regression.
2. The gradient boosted tree was chosen *specifically* because it is known as one of the best “off-the-shelf” machine learning techniques that requires little to no hyper-parameter specification or tuning. We experimented with different “cut-rates” for diagnosis codes and found little variance in model performance within our experimental range. The “cut-rate” is used to preserve those codes whose frequency of occurrence was above a threshold and the experimental range we used was: [10, 20, 40, 60, 80, 100]. Model performance began to decrease as we reach the 80-100 mark. The XGBoost classifier was called using default learning parameters with exception of the learning rate, which was set lower than the default of 0.1 to prevent overfitting. We have added this detail to (page 7, middle of page).
3. As a result of the reviewer’s feedback we have added detail to and rewritten sections of (pages 6 and 7) in order to convey the motivation and the implementation for both the feature-set and the training parameters we used.
4. We feel that the details regarding the GloVe embeddings and the second ANN are sufficient. We have added motivation for GloVe on (page 8, middle of page) to strengthen the clarity of the methods. We write:

*“The purpose of exploring GloVe embeddings and their potential impact on predictive readmission models is to discover if radical changes from current practices in feature-space and model design impact risk-standardization scores.”*

We hope these changes provide greater detail to the reader. Note that the code, but not data, is freely available: <https://github.com/wenshuoliu/DLproj/tree/master/NRD> (as is noted in the manuscript). Data are available to any readers through the NRD and AHRQ.

*2. The contribution of this work is not well-stated. It looks like that this paper is to recommend a method that combines medical code embedding and ANN for predicting readmission through the comparison. However, the clinical value of the proposed method in readmission prediction is not well-stated. Besides, the novelty of this paper should be addressed.*

**Response:** The reviewer is concerned about the contribution of our work and its clinical value. We now more clearly state why our findings are important relative to 2 reasons. First, a better understanding of differences in risk-standardization scores for readmission will help policymakers who are interested in documenting and understanding differences across hospitals. Medicare and other healthcare payers are quite interested in these issues and have paid out or withheld tens of millions in reimbursement already. Second, the ability of facilities to understand their performance can help guide the development and deployment of quality efforts that target readmission. It is important to note that neither of these reasons is related to “clinical” value. The use of administrative claims data for clinical insights is unlikely to be worthwhile, and we are careful not to state this throughout the paper. We believe a central contribution of this work is to nudge policy makers by showing two things: (1) it is possible to achieve better predictions using non-linear statistical models and (2) the use of these models leads to substantial changes in risk scores for hospitals. We have added some clarification throughout to strengthen these points.

As far as novelty goes, we agree that we have not emphasized enough the innovation of using medical code embeddings in this application. We now write in the Introduction:

*“Administrative claims data such as diagnosis code are key to describe a patient’s condition and other characteristics, but are often not in the easiest or most straightforward format for statistical analysis. We exploit a word embedding technique classically used in Natural Language Processing (NLP) to convert each diagnosis code into a numerical vector such that the “distance” between diagnosis codes is related to “semantic” similarity. Further, using these numerical embeddings as input features, we employ a newly developed deep set architecture ANN model to accommodate varying numbers of diagnosis codes across different patients and the fact that the prediction should be invariant with respect to the ordering of the diagnosis codes. ”*

This comment will better inform readers as to why tools like GloVe can help with analysis of diagnosis code in such models, and we believe help to improve their performance.

*3. In terms of your results, I don't think there is much difference in the comparison to show the superior of your proposed methods given the unclear experimental details. Suggest you can add other evaluation metrics such as PRC, sensitivity, F1 score.*

**Response:** The reviewer raises two issues related to the experimental results: (1) the reviewer states that the experimental details are unclear. (2) the reviewer has concerns about the appropriateness of the evaluation of model performance.

(1) **Clarity:** based on the reviewer’s comments we have added experimental details to the section ‘Statistical Models and Analysis’ starting on page 6. We have added details regarding the justification for the inclusion of the logistic regression model as a baseline comparison on page 7. We have added details regarding the training set and hyperparameter tuning procedures for the XGBoost model (page 7, paragraph 2). We have re-written the section on the ANN to provide greater details, including moving some material from the supplemental material to the body of the manuscript (page 8, paragraph 3). It is important to consider that even if ANN models performed predictively similarly, the resulting risk-standardization changes are still significant. Given that non-linear statistical measures perform “on-average” better than linear techniques that are currently used to calculate risk-scores, we argue that it would be valuable to consider these in the future and explore them as a potential option.
(2) **Choice of Metrics:** We have presented AUROC because it is threshold invariant and is increasingly commonly understood by a wide audience (especially health policymakers that we are targeting in this work). Further, we have included a *model* trained on the same instances (though different information) as a way to validate the performance differences. We are confident that the use of logistic regression as a baseline is appropriate to both the machine learning and policy-maker communities and have made an effort to clarify this point throughout.

*4. I am also quite confused about your comparisons.
If my understanding is correct, the 4 comparisons in your paper are 1. LR+comorbidity index 2.XGBoost+dummy variables 3.ANN+ dummy variables 4.ANN+ embedding. I would suggest you should add XGBoost+comorbidity index, ANN+comorbidity index, XGBoost+embedding, LR+dummy variables to fully justify the superior of embedding and ANN that you recommended.*

**Response:** The reviewer asks for greater clarification over the comparisons presented. We have attempted to clarify not only the details of our current choices, but also the motivations of our choices throughout in response to reviewer feedback (see earlier points that were covered above under Reviewer 1). We re-iterate, that predictive performance is only one of two purposes of this work (and arguably the less important contribution). The model comparisons that the reviewer identifies are correct, and their concise motivations are as follows:

1. LR + comorbidity index: develop an approximation of current practices and a reasonable performance baseline.
2. XGBoost +dummy variables: A sophisticated “off-the-shelf” machine learning approach given *more data* than a logistic regression model.
3. ANN + dummy variables: a state-of-the-art statistical learning technique that is fundamentally different from tree-based function approximation given *more data* than a logistic regression model.
4. ANN + embedding: a state-of-the-art statistical learning technique in combination with a numerical embedding technique frequently used in natural language processing.

The reviewer suggests new comparisons to those presented in the manuscript. We are excited by this as a viable direction for future research, but feel that this is not in the scope of the current work. However, preliminary results show that XGBoost and ANN are non-performant (with AUCs ~0.5) when trained on comorbidity index. This makes intuitive sense because the comorbidity index loses much information contained in the original diagnosis code. It should be noted that XGBoost can not be trained using embeddings as different patients may have different number of diagnosis and procedure codes. One advantage of the deep set ANN architecture that we employed is to accommodate different numbers of diagnosis codes across different patients. It should also be noted that LR + dummy variables (suggested by the reviewer) is a special of ANN + dummy variables (when there is no hidden layer in ANN), and we did find that ANN with hidden layers predicts better than without hidden layers (i.e. LR) in this particular problem.
