# Supplementary material for: Predicting 30-day hospital readmissions using artificial neural networks with medical code embedding
Source: PLoS One. 2020 Apr 15;15(4):e0221606. doi: 10.1371/journal.pone.0221606 (PMC7159221; doi:10.1371/journal.pone.0221606)
Supplement: Attachment — Submitted filename: _RESPONSE_LETTER_3-2-2020.docx [file pone.0221606.s009.docx]

**Please see our responses to the reviewer comments in red below.**

**REVIEWER COMMENTS**

1. If the authors have adequately addressed your comments raised in a previous round of review and you feel that this manuscript is now acceptable for publication, you may indicate that here to bypass the “Comments to the Author” section, enter your conflict of interest statement in the “Confidential to Editor” section, and submit your "Accept" recommendation.

Reviewer #1: All comments have been addressed

Reviewer #2: All comments have been addressed

2. Is the manuscript technically sound, and do the data support the conclusions?

The manuscript must describe a technically sound piece of scientific research with data that supports the conclusions. Experiments must have been conducted rigorously, with appropriate controls, replication, and sample sizes. The conclusions must be drawn appropriately based on the data presented.

Reviewer #1: Yes

Reviewer #2: Yes

3. Has the statistical analysis been performed appropriately and rigorously?

Reviewer #1: Yes

Reviewer #2: Yes

4. Have the authors made all data underlying the findings in their manuscript fully available?

The [PLOS Data policy](http://www.plosone.org/static/policies.action#sharing) requires authors to make all data underlying the findings described in their manuscript fully available without restriction, with rare exception (please refer to the Data Availability Statement in the manuscript PDF file). The data should be provided as part of the manuscript or its supporting information, or deposited to a public repository. For example, in addition to summary statistics, the data points behind means, medians and variance measures should be available. If there are restrictions on publicly sharing data—e.g. participant privacy or use of data from a third party—those must be specified.

Reviewer #1: Yes

Reviewer #2: No

**Response:** The Nationwide Readmissions Database (NRD) used for this study is a repository from Healthcare Cost and Utilization Project (HCUP), which is maintained by the Agency for Healthcare Research and Quality (AHRQ). We have provided a direct link to the HCUP website in the manuscript where researchers can take steps to access the third-party data used in our analysis. The link was included in all previous submissions as is on (page 4, line 232) of the revision attached:

<https://www.hcup-us.ahrq.gov/tech_assist/centdist.jsp>

We are bound by a Data Use Agreement with HCUO that forbids us from republishing the data.

5. Is the manuscript presented in an intelligible fashion and written in standard English?

PLOS ONE does not copyedit accepted manuscripts, so the language in submitted articles must be clear, correct, and unambiguous. Any typographical or grammatical errors should be corrected at revision, so please note any specific errors here.

Reviewer #1: Yes

Reviewer #2: Yes

6. Review Comments to the Author

Please use the space provided to explain your answers to the questions above. You may also include additional comments for the author, including concerns about dual publication, research ethics, or publication ethics. (Please upload your review as an attachment if it exceeds 20,000 characters)

Reviewer #1: All comments have been sufficiently addressed in the revised manuscript. No further comments on the revision.

Reviewer #2: Thank you for addressing my comments. Methods are not very innovative but reasonable. The data and analysis could support the conclusion. The manuscript is well-written.
Some minor problems: there might be typos or document disorder as I cannot find corresponding SFigures in the supporting document (they are SFigure or eFigure?). Authors should recheck it. Besides, I would suggest more explanation/clarification for the Figure 1 (such as adding axis title)

**Response:** We thank the reviewer for a careful review of the manuscript.

Regarding Figure 1. We have added two additional sentences to the caption to guide readers in interpreting distributions of risk-adjusted readmissions rates (page 12, line 502). We have also added axis labels to Figure 1.

Regarding supplemental figures (SFigures): we have included a separate revision of the supplemental material addressing figure labels. All attached figures are correctly labeled in the manuscript and in supplementary material.

7. PLOS authors have the option to publish the peer review history of their article ([what does this mean?](https://journals.plos.org/plosone/s/editorial-and-peer-review-process#loc-peer-review-history)). If published, this will include your full peer review and any attached files.


If you choose “no”, your identity will remain anonymous but your review may still be made public.


**Do you want your identity to be public for this peer review?** For information about this choice, including consent withdrawal, please see our [Privacy Policy](https://www.plos.org/privacy-policy).

Reviewer #1: No

Reviewer #2: No
